# Connecting the Dots: Mitochondrial Dysfunction, PCOS, and Insulin Resistance—Insights and Therapeutic Advances

**DOI:** 10.3390/ijms26136233

**Published:** 2025-06-28

**Authors:** Samia Palat Tharayil, Pallavi Shukla

**Affiliations:** Department of Molecular Endocrinology, The Indian Council of Medical Research-National Institute for Research in Reproductive and Child Health (ICMR-NIRRCH), J.M. Street, Parel, Mumbai 400012, India

**Keywords:** polycystic ovary syndrome, insulin resistance, ROS, mitochondrial dysfunction, hyperandrogenism

## Abstract

Insulin resistance (IR) frequently develops in women with polycystic ovary syndrome (PCOS), an endocrinological disorder typified by hyperandrogenaemia, erratic menstrual cycles, and the presence of multiple cysts in the ovaries. It results in elevated androgen production contributing to the clinical manifestations of the syndrome including associated co-morbidities such as obesity and type 2 diabetes (T2D). Mounting data suggest the involvement of free fatty acids, reactive oxygen species (ROS) signalling, and mitochondrial dysfunction with IR. In recent years, numerous reports have suggested that mitochondrial dysregulation is associated with the pathogenesis of PCOS. Increased ROS, mutations/variants in mitochondrial DNA (mtDNA), and the altered expression of nuclear-related mitochondrial genes in insulin-resistant women with PCOS provide sufficient evidence for mitochondrial dysfunction as one of the factors contributing to PCOS pathogenesis. Despite the advancements in the field of interconnecting links between mitochondrial dysfunction, IR, and PCOS, various underlying mechanisms needs to be elucidated. Advancements in therapeutic interventions showed promising results in improving mitochondrial functions and IR in PCOS pathogenesis, including evolving mitochondrial transfer approaches that may improve in vitro fertilisation (IVF) outcomes in obese and insulin-resistant women with PCOS in future.

## 1. Introduction

Polycystic ovary syndrome (PCOS) is an endocrine dysfunction prevalent globally in 6–12% of women in their childbearing years. It is marked by erratic menstrual cycles, heightened androgen secretions, and the presence of multiple cysts in the ovaries. PCOS also predisposes women to many co-morbidities, in particular, obesity, metabolic syndrome (MetS), type 2 diabetes (T2D), cardiovascular diseases (CVDs), and endometrial cancer (EC) over time [1,2,3]. While the exact contributors of PCOS are still not fully characterised, insulin resistance (IR) and bad lifestyle/dietary factors coupled with genetic predisposition has been identified as a significant determining factor [4]. Alterations in insulin receptor structure or function, leading to aberrant signalling pathways or heightened levels of insulin-binding antibodies, reduce the sensitivity of peripheral tissues to insulin, leading to IR [5]. Furthermore, factors such as obesity exacerbate IR and are implicated in the metabolic syndrome, a frequently observed phenomenon in PCOS [6]. Obstructive sleep apnoea (OSA) and depression observed in women with PCOS are associated with increased activity in the sympathetic nervous system. OSA is related to hyperinsulinemia and is exacerbated by obesity. OSA may accelerate PCOS symptoms and is related to metabolic and CVD in these women [4]. Weight gain is becoming more prevalent among women with PCOS, with rates reaching up to 88% [7,8]. A different study indicates that 75% of lean and 95% of obese women diagnosed with PCOS experience IR [9]. Moreover, reduced insulin sensitivity inevitably results in hyperinsulinemia, which in turn fosters the development of hyperandrogenism by exerting a chronic stimulus on the cells of the ovarian theca [10].

In women with PCOS, the aberrant secretion patterns of gonadotropins such as luteinizing hormone (LH) and follicle-stimulating hormone (FSH), as well as ovarian steroids, contribute to IR [11]. Insulin, in turn, stimulates androgen synthesis and enhances LH function. This results in the overproduction of testosterone, androstenedione, and dehydroepiandrosterone by the ovaries in PCOS patients, as demonstrated in previous study [12]. It is plausible that hyperandrogenism plays a significant part in the pathogenesis of serious endocrine and metabolic disturbances linked to PCOS. Women diagnosed with PCOS having reduced sex hormone-binding globulin (SHBG) levels are more prone to obesity, IR, T2D, hyperandrogenism, and cardiovascular disease (CVD) [13], primarily owing to prevailing IR [14,15].

Due to IR, women with PCOS have elevated blood sugar levels that put mitochondria under non-physiological stress, resulting in excessive reactive oxygen species (ROS) production. Moreover, mitochondrial DNA (mtDNA) lies in the proximity of electron transport chain (ETC) is susceptible to considerable damage due to oxidative stress (OS). In addition, due to a lack of efficient repair mechanisms, these mtDNA mutations can pile up and result in metabolic changes. Evolutionarily speaking, many loci in mitochondrial genome are conserved and cause small genetic variants to have significant ramifications in the pathogenesis of PCOS [16].

In this review, our aim is to reinforce the connections between insulin resistance, PCOS, and mitochondrial dysfunction by highlighting the robust association between PCOS and IR, emphasizing the role of mitochondrial dysfunction in both IR and PCOS, and this is supported by evidence of mtDNA variants associated with IR in PCOS. Additionally, we showcase advancements in therapies developed over the years to address mitochondrial dysfunction/OS and IR in women with PCOS.

## 2. Mitochondrial Biology

Mitochondria are double membrane-bound organelles containing a small circular genome (mtDNA) that is present in multiple copies in the cell [17,18]. There are roughly one to ten copies of mtDNA in each mitochondrion, with around 16,569 base pairs which codes for a displacement loop (D-Loop), 22 tRNAs, 2 rRNA, and 13 polypeptides required for RNA and protein synthesis [19,20]. The 13 polypeptides that are specified by mtDNA are subunits of complexes I through V, forms the mitochondrial respiratory chain are crucial for OXPHOS ATP synthesis [21]. D-loop, a non-coding sequence of mtDNA, contains regulatory components necessary for mtDNA transcription and replication [22]. The majority of the proteins of OXPHOS subunits are encoded by nuclear DNA, aside from their own genetic material. Compared to nuclear DNA, mtDNA is more prone to damage and mutation because it is not shielded by histones [18]. Mitochondrial biogenesis involves the coordinated synthesis of new proteins by both the nucleus and existing mitochondria to expand the cellular network of mitochondria. This process typically escalates in response to heightened energy requirements, such as during cellular proliferation. Furthermore, it could also ramp up to replace mitochondria damaged by the stressors of environment or OS [23]. Peroxisome proliferator-activated receptor-gamma co-activator α (PGC-1α), the principal regulator of biogenesis of mitochondria, is activated by phosphorylation in the nucleus stimulates string of transcription factors such as nuclear respiratory factor-1 (NRF-1), NRF-2, oestrogen-related receptor-a (ERR-a), and transcription factor A (TFAM). Out of these, NRF-1 enhances the transcription of nuclear genes responsible for controlling mitochondrial function, while NRF-2 boosts the transcription of genes crucial for redox homeostasis [24]. PGC-1 therefore increases the mitochondrial mass, as well as the capacity of mitochondrial oxidative phosphorylation [17]. TFAM is another factor that transcribes and replicates mtDNA [24]. Its expression is also regulated by NRF-1 [25]. However, the equilibrium of the cellular environment is maintained by a process known as mitophagy, which removes damaged mitochondria and its absence can result in a high number of dysfunctional mitochondria, impairing overall energy production. Furthermore, the accumulation of dysfunctional mitochondria can lead to the generation of ROS, triggering inflammation and signalling cell death. Generally, defective mitochondria are cleared via PTEN-induced putative kinase 1 and parkin-mediated mitophagy to control mitochondrial quality [17]. Mitochondria also have the ability to undergo both fusion and fission processes in order to sustain their functionality [26]. Mitochondrial fusion combines their contents, including DNA and metabolic substances, aiding in the restoration of damaged or depolarised membranes [27]. Three GTPases, namely mitofusin 1 and 2 (Mfn1/2) and optic atrophy1 (Opa1), situated on the outer and the inner membrane of the mitochondria, respectively, predominantly regulate this process [25,28,29]. The fusion of the outer membrane is mediated by mitofusin 1 and 2, while the fusion of inner membrane utilises optic atrophy protein [17]. Meanwhile, mitochondrial fission is regulated by the activity of Fis1 and dynamin-related protein 1 (DRP1), located on the outer membrane, which augment the quantity of mitochondria, preparing the cell for cellular division and meiosis [30]. Furthermore, mitochondria are also involved in the Ca^2+^-based signalling essential for diverse processes ranging from ATP production to cell death [31].

## 3. Role of Free Fatty Acids, ROS Signalling, and Mitochondrial Dysfunction in IR

**Figure 1:**
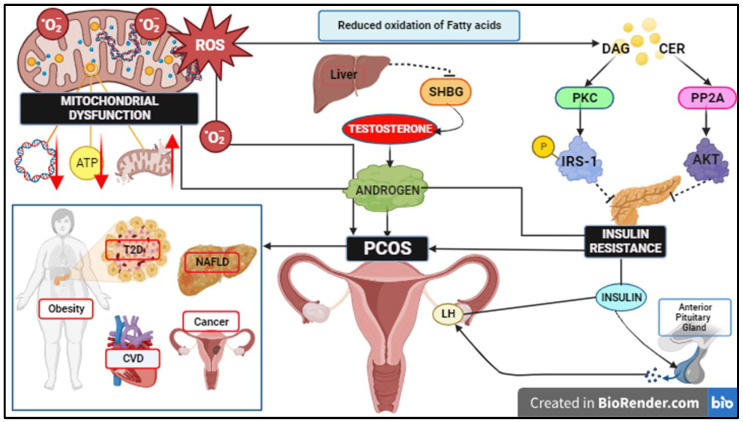
Diagram illustrating the complex interplay between mitochondrial dysfunction, PCOS, and IR. DAG and CER link mitochondrial dysfunction to IR by activating protein kinase C and inhibiting AKT, respectively, and through increased ROS production. Insulin acts as a co-gonadotropin and enhances LH effects on androgen biosynthesis in ovarian theca cells. Excess insulin inhibits SHBG-release from the liver, increasing free testosterone levels and leading to hyperandrogenaemia, a clinical manifestation of PCOS. IR predisposes women with PCOS to comorbidities such as obesity, CVD, T2D, NAFLD, and EC.

An interdependent relationship exists between insulin and mitochondria, as insulin is required for mitochondrial fusion and mitochondria are needed to ensure proper insulin signalling [32,33]. Until now, two proposed mechanisms associate mitochondrial dysfunction with IR. One includes the partial oxidation of fatty acids, which contributes to fatty acid metabolites accumulation, resulting in the inhibition of insulin signalling and the other mechanism involves elevated ROS production due to electron leakage, a consequence of incomplete substrate oxidation. This impacts OS, leading to mitophagy and apoptosis, which, in turn, causes decreased substrate oxidation [34,35,36]. The first mechanism involves the incomplete oxidation of fatty acids such as diacylglycerol (DAG) and ceramide (CER), forming a plausible link between mitochondrial dysfunction and IR, as these supress insulin signalling by activating protein kinase C, which is translocated to the plasma membrane, resulting in insulin receptor [37] and protein kinase B (AKT) inhibition, respectively (Figure 1), [25,34,38]. The kinases activated by these lipid metabolites particularly impair insulin receptor substrate 1 (IRS1) [31,39]. In skeletal muscle, this impairment leads to a decrease in glucose transporter protein type-4 (GLUT4) expression and a subsequent decrease in cellular glucose uptake, further hampering the insulin signalling process [40]. Ectopic lipid build-up is predominantly seen in obesity and T2D as a particular initial change in the onset of IR [25,38]. The incompetency to store surplus energy in adipose tissue leads to an increased outflow of free fatty acids (FFAs) from fat stores into other tissues, such as skeletal muscle [41], causing metabolic dysregulation, including IR. Insufficient fatty acid oxidation resulting from mitochondrial dysfunction and/or decreased mitochondrial content further accumulates elevated levels of intracellular fatty acyl-CoA and DAG, which interfere with insulin signalling [42,43]. A study reported that in the adipose tissue of obese mice fed with a high-fat diet, reduced Mfn1/2 expressions and elevated Drp1 were observed, respectively. However, in the adipose tissue of obese humans only Mfn2 reduction was observed [44]. Moreover, in the skeletal muscle, an excess of FFA uptake increases the rate of beta-oxidation in obese individuals, causing mitochondria to become shorter and smaller due to heightened mitochondrial fission, thereby reducing its mass and function [45,46]. In another study, excess palmitate (PA) induced an increase in DRP1 and FIS1 (fission proteins) and the fragmentation of mitochondria in differentiated C2C12 muscle cells. Fragmentation resulted in heightened OS, mitochondrial dysfunction, and diminished insulin-stimulated glucose uptake [43]. Furthermore, it has been reported that peptides released during stress condition by mitochondria, such as apoptosis-inducing factor (AIF), impact insulin action. AIF is a peptide that forms part of complex I of ETC; when released from mitochondria, it induces chromatin condensation and DNA fragmentation in response to apoptotic signals [47]. Knocking out AIF in both the liver and muscles shielded mice from developing IR when exposed to a high-fat diet [48]. The impact of AIF deletion during insulin activity may occur through mechanisms akin to those of anti-diabetic drugs which inhibit complex I, or AIF might possess unrecognised signalling roles that affect cellular insulin activity [31]. Improvements in insulin sensitivity could be restored by increasing lipid oxidation, conferring protection against IR [40,49]. It has been reported that high glucose and FFAs increase the ROS generation by activation of NADPH oxidase 4 (NOX4), and their inhibition, along with monocyte chemotactic protein-1 (MCP-1) expression in differentiated adipocytes, reduces ROS production [50,51]. Moreover, the adipocyte-specific deficiency of NOX4 delays the onset of adipose inflammation and IR [52]. Meanwhile, the overexpression of NOX4 in excess overnutrition significantly reversed the inhibition of protein tyrosine phosphatase 1B (PTP1B), contributing to IR in adipocytes [46,53].

The result of incomplete substrate oxidation, which affects electron flow via the ETC and contributes to the generation of superoxide anions by reacting with molecular oxygen, is another plausible mechanism that connects mitochondrial dysfunction to IR. ROS generation damages various mitochondrial components such as DNA, proteins, and lipids [25]. In addition, the leakage of H_2_O_2_, which can readily pass the mitochondrial membrane, reaches these components, which, in the presence of Fe^2+^ ligands, can further generate OH radicals [43]. These produced OH radicals contribute to the inactivation of the components of enzymes involved in the tricarboxylic acid (TCA) cycle [43]. ROS initiates cellular signalling by modifying the thiol groups of the cysteine residues of the target protein, resulting in changes to the protein structure [43,54] and contributing to the overall OS [25]. Moreover, the elevated ROS levels enhance the phosphorylation of insulin receptors and IRS proteins and incite the aberrant activation of serine/threonine kinase signalling pathways, including c-Jun N-terminal kinase (JNK), nuclear factor kappa-B (NF-kB), and p38-mitogen-activated protein kinase (MAPK) [55]. Additionally, ROS also suppresses GLUT4 translocation in cells by affecting insulin signalling [56]. It is also known that insulin ensures proper mitochondrial functioning by maintaining NAD^+^/NADH ratio in ETC integrity by suppressing forkhead box 01 (FOXO1) and heme oxygenase-1 (HMOX1) [33,57]. It was also found that cultured cardiac muscle cells exposed to elevated glucose levels trigger the activation of NADPH oxidase 2 (NOX2), brought about by the activation of Ras-related C3 botulinum toxin substrate 1 (RAC1) and the translocation of the 7 kDa cytosolic subunit of NADPH oxidase (P47PHOX), leading to the production of ROS [58].

Apart from the aforementioned mechanisms, other factors involved in IR-associated mitochondrial dysfunction include altered calcium buffering capacity, the reduced expression of mitochondrial-associated ER membranes (MAMs), oxidative enzymes, ATP surplus, and sirtuin 1 (SIRT1) activity. Mitochondrial dysfunction alters Ca^2+^ buffering capacity, which is required for the activation of calcium/calmodulin kinase II (CaMKII); this in turn phosphorylates myosin-1c (Myo1c), which aids in transport of GLUT4 to the plasma membrane in response to insulin stimulation in adipocytes [59]. Altered Ca^2+^ levels could affect insulin-stimulated CaMKII activity and glucose transport action in these cells [31]. The points of physical connection between mitochondria and the endoplasmic reticulum (ER) are referred to as MAMs. The increased expression of MAM proteins demonstrated enhanced insulin sensitivity in the liver [60] and reduced the levels of cyclophilin-D, a mitochondrial protein, resulting in systemic IR and elevated blood glucose levels after administering pyruvate, which suggests heightened hepatic glucose production [60,61]. Additionally, inhibiting cyclophilin-D or removing MAM content disrupted the Ca^2+^ exchange between ER and mitochondrial cyclophilin-D knockout mice, leading to liver ER stress and mitochondrial dysfunction [62,63]. It has also been noted that IR follows ATP surplus, or the state in which ATP synthesis surpasses demand, in a number of tissues [64], leading to hyperinsulinemia and hyperglucagonemia. In order to reduce insulin sensitivity, it also activates and inhibits the mammalian target of rapamycin (mTOR) and AMP-activated protein kinase (AMPK) signalling pathways, respectively. Moreover, IR is facilitated by the decrease in AMPK activity brought on by mitophagy suppression [65]. According to a further study, individuals with T2D and insulin resistance with normal glucose tolerance have reduced expression of PGC-1α and 1ß [42,66,67]. Patients with T2D who were also obese showed a decreased activity of several mitochondrial oxidative enzymes [68,69]. Recently, it was observed that mitochondrial DNA methyltransferase 1 (DNMT1) and NAD^+^-dependent deacetylase SIRT1 have strong associations with IR [70,71,72]. It was shown that DNMT1 could be deacetylated by SIRT1, thus regulating the gene expression [73,74,75]. According to reports, insulin-resistant individuals have a considerably lower SIRT1 gene and protein expression in peripheral blood cells compared to insulin-sensitive individuals [72]. Furthermore, IR was shown to lower SIRT1 activity and cellular NAD^+^ levels in vivo [71,76].

## 4. IR in the Pathophysiology of PCOS

IR affects 38% to 95% of women diagnosed with PCOS, which is independent of obesity [36]. A study suggested that the impact of subcutaneous abdominal fat and visceral fat on IR differed, with subcutaneous fat protecting against IR and visceral fat causing IR by elevating FFA uptake in visceral adipocytes, in addition to non-adipose cells inducing lipotoxicity [77]; this would explain the incidence of lean women with PCOS and IR [18]. It is well established that PCOS is characterised by elevated testosterone and androstenedione levels, contributing to hyperandrogenism [78]. There are different mechanisms by which hyperinsulinemia aids androgen-induced anovulation. Insulin acts as a co-gonadotropin [79] and stimulates the effects of LH [80,81] on androgen biosynthesis in ovarian theca cells by inducing cytochrome P450c17 expression, which influences 17-hydroxylase and 17,20-lyase activity [56], making theca cells in women with PCOS more sensitive to the hyperandrogenic effects of insulin in contrast to healthy women [82]. Alternatively, there have been studies conducted in vivo and in vitro [83,84] which report that an excess of insulin inhibits SHBG release from the liver, thus elevating androgen levels [85] (Figure 1). This modification of SHBG levels enhances the bioavailability of free testosterone in the blood, thereby triggering heightened androgenic activity [85]. In addition to insulin, the anti-mullerian hormone (AMH), which is typically elevated in PCOS, also stimulates gonadotropin hormone-releasing hormone (GnRH) neurons, potentially promoting hyperandrogenism. Research indicates that daughters born to mothers with PCOS could potentially inherit a susceptibility due to abnormal exposure to androgens and AMH during gestation [85].

In a different study [86], women with PCOS exhibited lower kisspeptin serum levels, a peptide involved in puberty activation and GnRH pulsatile secretion during ovulation, when compared to controls. In addition, among PCOS subjects, the overweight or obese group exhibited lower levels of kisspeptin with negative correlations with homeostatic the model assessment of insulin resistance (HOMA-IR), body mass index (BMI), and androgens, thus suggesting that IR plays a role in reducing kisspeptin levels [87]. Although the association between IR and BMI in PCOS subjects remains divisive, it has been reported that non-obese PCOS women could have elevated IR regardless of BMI [88], whereas others suggest an association between IR and BMI [77,89,90]. IR and hyperandrogenism are also caused by inflammation due to the death of hypertrophic adipocytes [91,92]. IL-6 is also reported to reduce the activation of IRS1 and AKT [78]. Single nucleotide polymorphisms (SNPs) have been identified in proinflammatory cytokines genes, such as tumour necrosis factor (TNF), IL-6 [93], IL-10 [94], IL-17, and IL-32 [95], and act as a genotypic-specific predisposition to PCOS. Hormone leptin levels are also elevated in PCOS subjects, which in turn upregulate interferon–gamma (INF-gamma) and IL-6 production by binding with insulin receptor [96]. Meanwhile, adipokine omentin-1 [97] levels are lower in women with PCOS and IR [77,89,90]. Furthermore, the reduced oxidation of cortisol could inhibit the insulin signalling pathway via PTEN induction expression in the epithelial cells of endometrium, thus contributing to IR in women with PCOS [78]. Furthermore, as evidenced by elevated lipolysis and P53 activation in adipose tissue in an animal model of heart failure [98], the sympathetic nervous system (SNS) is also reported to be dysfunctional when coupled with IR. This implies that by promoting SNS dysfunction, IR fuels inflammation in PCOS [72]. Women with PCOS are more susceptible to several comorbidities, for instance MetS T2D, CVD, non-alcoholic fatty acid liver disease (NAFLD), and (EC) (Figure 1) [18]. IR plays a pivotal role in the pathogenesis of MetS [99], which is prevalent in 50% of women with PCOS [100]. Additionally, androgens also contribute significantly to the disease progression of MetS, with higher percentages being recorded in hyperandrogenic PCOS women (24.8%) compared to the non-hyperandrogenic counterparts. Strikingly, both waist circumference and free androgen index are independently correlated with MetS and IR [99]. Unfortunately, distinguishing the effects of obesity from MetS in women with PCOS is quite challenging [101]. IR is also a major mediator which further intensifies the risk of the metabolic disorders such as T2D and CVD [18] as it decreases nitric oxide (NO) and increases endothelin-1 (ET-1) in arterial endothelial cells. The increased synthesis of vasoconstrictors further impairs the vasodilation of insulin [102]. IR elevates the hepatic secretion of very-low-density lipoprotein (VLDL), eliminates VLDL and chylomicrons from circulation, and enhances the clearance of the high-density lipoprotein cholesterol (HDL-c) component, apolipoprotein A (apoA) [103]. Women with PCOS have predominant lipid abnormalities with a prevalence of 70% and higher concentrations of oxidised low-density lipoprotein cholesterol (LDL-c), increasing the risk of CVD. Studies revealed that lipoprotein A (24%) and apolipoprotein B (apoB) (14%) levels are elevated in PCOS patients [99,104,105]. Additionally, the decreased inhibition of lipolysis in adipose tissue, which results in an increased influx of FFAs into the liver and steatosis, is a function of IR [106], leading to NAFLD [107]. It has been speculated that hyperandrogenism impacts the expression of the LDL receptor (LDLR) in both adipocytes and liver, which is downregulated by 0.51-fold in women with PCOS [100,108]. Androgen excess in PCOS leads to the hypertrophy of adipocytes, and both are related to IR [55]. Moreover, inflammation is also associated with adipocyte hypertrophy, resulting in vascular compression, hypoxia, and increased inflammatory markers [55]. In ovaries, inflammation stimulates steroidogenic activity, theca cell proliferation, and the phosphorylation of the receptor, further increasing androgen excess and IR leading to the development of comorbidities such as T2D, atherosclerosis, and hypertension in women with PCOS [55]. The higher incidence of IR in women with PCOS also predisposes them to increased exposure of EC [109]. The increased expression of the insulin receptor along with the insulin-like growth factor 1 (IGF-1) receptor in endometrium leads to the crosstalk of their signalling pathways, contributing to the development of EC in women with PCOS [107,110].

## 5. Mitochondrial Dysregulation in the Pathophysiology of PCOS

Women with PCOS have compromised mitochondrial structures, content and dynamics in circulating leucocytes [111,112,113]. In addition to PCOS, altered mitochondrial morphology is also observed in other metabolic diseases, neurodegenerative disorders, and cancers [114,115]. ROS is the main pathogenesis factor in PCOS, as it is directly linked to mtDNA and OXPHOS efficiency (Figure 1). The marker levels of ROS in circulation, such as malondialdehyde (MDA), superoxide dismutase (SOD), and glutathione peroxidase (GPx), are heightened in women with PCOS, as the generated ROS overwhelms the antioxidant defence system. ROS also interferes with several cellular processes such as cell growth, differentiation, proliferation, and apoptosis [116]. As mtDNA is characterised as a biomarker for mitochondrial impairment, assessing its quantity serves as ideal objective in women with PCOS [24]. Lee et al. found a reduced mtDNA copy number in women with PCOS [117]. Similarly, in another study, women with PCOS and IR who also harboured mt-tRNA mutations were reported to have a lower number of mtDNA copies [118]. 

In the follicular fluids of PCOS patients, increased MDA levels, lowered total antioxidant capacity (TAC), and decreased thiol concentrations have also been reported. These results are also further reinforced by the analysis of ROS production in granulosa cells and leucocytes by Lai et al., where it was found that granulosa cells exhibited four times higher ROS compared to those of controls [90,119]. In another study, changes in the level of TCA and NAD catabolism in the follicular fluid, along with OS in cumulus cells (CCs), demonstrated a downregulated mitochondrial biogenesis rate, mtDNA, MMP, and *PGC-1α* gene expression and an upregulated *PGC-1α* promoter methylation rate in women with PCOS, compared to control group CCs [55]. Evidence of mitochondrial dysfunction in PCOS extends to broader reproductive biology, with the identification of 30 genes contributing to mitochondrial dysfunction signalling pathways in the granulosa cells of primordial follicles, including *ATP5H*, *NDUFA2*, *PSEN2*, and other apoptosis-regulating genes [120,121]. Furthermore, a metabolomics-based study of follicular fluid in PCOS patients revealed that mitochondrial dysfunction in granulosa cells, redox imbalance, and elevated OS levels may partly explain the metabolic disorders seen in PCOS [122].

Mitochondria could essentially be the target organelle that hampers the metabolic energy in obese PCOS patients who predominantly have IR. Severe OS can trigger obesity through the proliferation of preadipocyte followed by differentiation, thereby enlarging the mature adipocyte [123] and signalling hypothalamic neurons to increase hunger [55,124]. In addition, mitochondrial dysfunction and OS also contribute to inflammation and IR in PCOS. The formation of ROS by mononuclear cells has been linked to the aberrant development of pancreatic kb cell function, according to Malin et al. [125]. This is because ROS-induced OS triggers the activation of a nuclear protein that binds to DNA and promotes the transcription of TNF α [126]. According to observations, the creation of TNF diminishes the mtDNA-encoded cytochrome c oxidase 1 (COX-1) subunit, which lowers intracellular ATP synthesis and increases ROS accumulation, aggravating IR [121,127]. In a different study, myeloperoxidase (MPO), an enzyme released by WBC in inflammatory sites, and ROS production were assessed in PCOS-associated IR and non-insulin-resistant PCOS patients, with elevated levels being observed in women with PCOS-associated IR [55,128].

According to a further study, obese woman with PCOS and IR exhibited aberrant mitochondrial physiology in skeletal muscle, reduced phosphorylation efficiency, and elevated H_2_O_2_ emissions in contrast to insulin sensitive women [129]. Skov et al. studied the expression of OXPHOS genes in the skeletal muscle of women with PCOS and identified reduction in OXPHOS coupling efficiency in the obese (BMI > 33 kg/m^2^) women with elevated H_2_O_2_ emissions as compared to lean (BMI < 23 kg/m^2^) women [130]. These conditions bolster the compromised mitochondrial bioenergetics in obese women due to OS [55,129,130,131]. However, in skeletal muscle biopsies, no difference in mtDNA copy number was observed between the PCOS and control groups [132].

Moreover, several nuclear encoded OXPHOS genes, such as *NDUFA3*, *SDHD*, *UCRC*, *COX7C*, and *ATP5H*, were identified to be downregulated in women with PCOS [55]. *NDUFA3* expression, which plays a role in follicular development and IR, is also associated with mtDNA copy number [133]. A further study observed that the SNPs in *SDHD* correlated with BMI, thus increasing obesity risk [134]. Meanwhile, the knockdown of *COX7C*, a gene inhibited during obesity, led to the accumulation of myocardial fat [55].

In another study, an induced pluripotent stem cell (iPSC) derived from the somatic cells of women with PCOS exhibited higher mtDNA copy numbers, biogenesis, and impaired respiration function, along with a higher expression of *PGC1α*, *TFAM*, and *NRF-1* when compared to non-PCOS-derived iPSCs. The expressions of GLUT1 and GLUT3 were also decreased, indicating their roles in IR [55].

## 6. Variants Associated with IR in mtDNA Genes and Nuclear-Related mtDNA Genes in PCOS

Mitochondrial dysfunction can be a result of variants present in mtDNA genes or in nuclear-encoded mitochondrial genes (Table 1). The D-loop region is the most widely studied for mutational analysis in mtDNA, including deletions that impair mitochondrial functions while nuclear related genes are least studied [56,135]. Several frequent occurring D-loop variants were found in women of south-Indian ethnicity with PCOS [136]. In another study, many common to rare mtDNA variants in the D-loop region, as well as other mtDNA regions, were reported in women with PCOS in Indian and Chinese populations using next-generation sequencing (NGS) [136,137]. However, although these alterations were associated with mitochondrial dysfunction, their association with IR was not indicated in the reports.

While mitochondrial variants in PCOS have been extensively studied, there is limited research on variants specifically associated with insulin-resistant women with PCOS. These mtDNA mutations/variants may pose a risk for T2D in women with PCOS at later stages of their life. Different mtDNA alterations have been reported in PCOS-associated with IR, and all of these have strong functional implications regarding the stability and function of mitochondria (Table 1). The most conserved mitochondrial mutation, *ND1* T3394C (p.Y30H), is linked with IR in women with PCOS [138,139]. The functional analysis of this mutation revealed that it affects the stability of ND1 subunit as well as the overall complex I assembly and its activity compromises MMP and ATP levels by increasing ROS production [56,140]. The *ND2* C5178A corresponds to the substitution of leucine to methionine at the 237th amino acid position (p.M237L), which has been found to be associated with longevity [141] and acute myocardial infraction [141] and has also been reported to reduce ATP, MMP, and SOD activity and increase in ROS, MDA, and 8-hydroxydeoxyguanosine (8-OHDG), suggesting mitochondrial dysfunction [56,142]. Another mutation that was observed in PCOS patients with IR was tRNA Ser C7492T [143], which, along with homoplasmic *ND5* T12338C (p.M1T), alters the highly conserved methionine by threonine shortening the chain by two amino acids [144]. The cybrid cell models further confirmed that T12338C decreased the stability of *ND5* chain, thereby affecting the overall activity of respiratory complex [145]. Another mutation at *ND5*, T12811C (p.Y159H), impacted its transmembrane structure and function [146], suggesting a similar effect [56].

Furthermore, Ding et al. identified nine mt-tRNA-based mutations related to IR in women with PCOS, namely tRNALeu (UUR) C3275T, A3302G, tRNAGln T4363C, T4395C, tRNACys G5821A, tRNASer (UCN) C7492T, tRNAAsp A7543G, tRNALys A8343G, and tRNAGlu A14693G [118]. A homoplasmic tRNALys-based mutation found to be associated with IR in women with PCOS, A8343G has been reported to affect the adenine in TψC loop at the 54th position, impacting aminoacylation and binding affinity with mitochondrial elongation factor Tu, subsequently hampering the protein synthesis [56,147,148]. A sequencing analysis of a Chinese PCOS patient with IR identified a heteroplasmic tRNA Leu (UUR) A3302G mutation. This mutation occurred in the highly conserved region of the 5′ end of tRNALeu and impacted the stability of tRNA, as observed in cybrids, leading to severe complex I and IV deficiencies. This mutation also caused the abnormal processing of RNA19, an intermediate consisting of mitochondrial 16S rRNA, tRNALeu, and *ND1* [149]. The mother and grandmother of this index case were diagnosed with T2D. In another family, a well conserved tRNALeu (UUR) C3275T-based mutation linked with IR in PCOS was reported. This mutation was also considered a risk factor for Leber’s hereditary optic neuropathy (LHON) and disrupted DNA base-paring (28A-46C), impairing the tRNA metabolism [56]. Saeed et al. analysed the mitochondrial tRNALeu (UUR) gene using Sanger sequencing (R = A or G), identifying ten mutations, with 80% located in the highly conserved region (3157–3275). These mutations (A > G and/or T > C) were predicted to disrupt secondary tRNA structure, affecting base pairing. Mutations that maintained base pairing were only observed in healthy controls. This highlights the role of tRNA mutations in the pathogenesis of IR associated with PCOS by impairing mitochondrial functions [150]. 

The analysis of mitochondrial D-loop identified variants, T152C, 523delAC, T16126C, and A16203G associated with increased or decreased IR in women with PCOS. Variants T16126C and A16203G were associated with IR (HOMA-IR > 4.86). In contrast, variants T152C and 523delAC were associated with non-IR (HOMA-IR ≤ 4.86) in women with PCOS [151].

**Table 1:** mtDNA and nuclear-related mitochondrial gene variants associated with IR in women with PCOS.

Gene	Locus	Mutation	Nucleotide Positions in tRNA/Protein Change	Type	Homoplasmy/ Heteroplasmy	Associated Disease	Reference
*tRNA Glu*	mtDNA	A14693G	54	Mutation	Homoplasmy	Deafness, HTN MELAS, LHON	[56,118,121]
*tRNA Lys*	mtDNA	A8343G	54	Mutation	Homoplasmy	MetS, PD risk factor, deafness	[56,118,121,152]
*tRNA Leu (UUR)*	mtDNA	C3275T	44	Mutation	Homoplasmy	MetS, LHON	[56,118,121,152]
*tRNA Leu (UUR)*	mtDNA	A3302G	71	Mutation	Heteroplasmy	MELAS, MM	[56,118,121,152]
*tRNA Gln*	mtDNA	T4363C	38	Mutation	Homoplasmy	HTN, MetS, possibly deafness, HTN, LHON	[56,118,121,152]
*tRNA Gln*	mtDNA	T4395C	6	Mutation	Homoplasmy	HTN	[56,118,121,152]
*tRNA Arg*	mtDNA	T10454C	55	Mutation	Homoplasmy	Possibly deafness, HTN	[56,118,121,152]
*tRNA Asp*	mtDNA	A7543G	29	Mutation	Heteroplasmy	MEPR	[56,118,121,152]
*tRNA Ser (UCN)*	mtDNA	C7492T	26	Mutation	Homoplasmy	CPEO, HTN, deafness risk factor	[56,118,121,152]
*NC7*	mtDNA	9-bp deletion		Deletion	Homoplasmy		[16,56,152]
*ND1*	mtDNA	T3394C	Y30H	Mutation		LHON, diabetes	[56,152]
*ND2*	mtDNA	C5178A	L237M	Mutation		Acute MI, Diabetes, atherosclerosis	[56]
*ND5*	mtDNA	T12338C	M1T	Mutation	Homoplasmy	EH, LHON, MIDD	[56,152]
*ND5*	mtDNA	T12811C	Y159H	Mutation	Homoplasmy	Possible LHON factor	[56,152]
*D-loop*	mtDNA	T16126C		SNP			[137]
*D-loop*	mtDNA	A16203G		SNP			[137]
*D-loop*	mtDNA	T16217C		SNP		Endometriosis	[151]
*D-loop*	mtDNA	A16316G		SNP			[151]
*D-loop*	mtDNA	A16203G		SNP			[151]
*PGC-1α*	nDNA	rs8192678	G482S	SNP		Risk factor for diabetes	[55,135]

CPEO: chronic progressive external ophthalmoplegia; EH: endolymphatic hydrops; HTN: hypertension; LHON: Leber’s hereditary optic neuropathy; MetS: metabolic syndrome; MIDD: mitochondrial diabetes and deafness; MELAS: mitochondrial encephalomyopathy;, lactic acidosis, and stroke-like episodes; MEPR: mitochondrial encephalomyopathy, pyruvate dehydrogenase deficiency, and recurrent episodes; mtDNA: mitochondrial DNA; MM: mitochondrial myopathy; MI: myocardial infarction; nDNA: nuclear DNA; PD: Parkinson’s disease.

Women with PCOS are also shown to have deletions based on mtDNA. A deletion frequently observed in PCOS subjects in the variable region of the mitochondrial genome is a 9 bp sequence (CCCCCTCTA). This deletion ascended from the errors caused by DNA polymerase–gamma, which lack repair mechanisms. Intriguingly, Hu et al. identified that these deletions were predominantly linked with elevated serum glucose levels and lower insulin sensitivity indexes, advocating the contribution of IR in PCOS [16].

Recently, a study on PCOS patients investigated the impact of D-loop polymorphisms on the association of HOMA-IR and HOMA-β with BMI. Variant T16217C enhanced the association between BMI and HOMA-IR, highlighting the potential role of the variant in exacerbating IR in PCOS patients; in contrast, variant A16203G reduced the association between BMI and HOMA-β. Variant A16316G weakened the association between BMI and both HOMA-IR and HOMA-β simultaneously, suggesting a protective effect by mitigating the adverse impact of increased BMI on both IR and beta-cell function [151]. 

Moreover, a different study demonstrated the role of the *PGC-1α* gene in predisposing individuals to IR. PCOS patients exhibited significantly different allelic frequencies and genotypic distributions of *PGC-1α* Gly482Ser polymorphism, with carriers of the *PGC-1α* rs8192678 “Ser” allele, resulting in a predisposition to developing PCOS [55,135].

## 7. Therapeutic Interventions Targeting IR and Mitochondrial Dysfunction to Ameliorate PCOS

**Figure 2:**
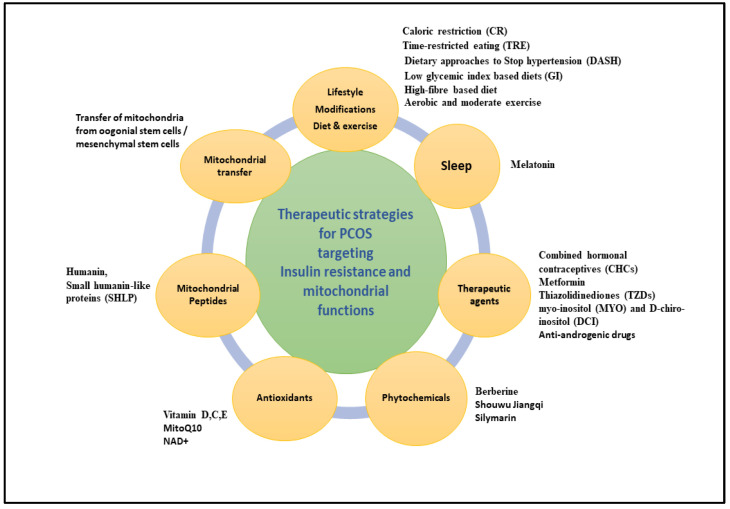
Schematic diagram illustrating diverse present and future therapeutic strategies targeting insulin resistance and mitochondrial dysfunction in PCOS pathophysiology.

Various strategies, ranging from lifestyle modification to therapeutic drugs, have been found to be beneficial in ameliorating IR and mitochondrial function in PCOS. Future therapeutic strategies, such as mitochondrial transfer, are under study (Figure 2).

### 7.1. Exercise

Regular exercise is considered a primary treatment option and lifestyle change in women with PCOS [153]. Exercise is associated with enhanced cardiorespiratory fitness, decreased waist circumference, improvement in insulin sensitivity, and sex hormone levels such as FSH and testosterone [154,155,156]. A study by Dantas et al. reported that exercise elicits increased phosphorylation at the 308th threonine residue of AKT and AMPK, suggesting efficient GLUT4 translocation in the skeletal muscle of women with PCOS [157]. In another study [158], the upregulation of IR-associated genes such as *PGC1α*, peroxisome proliferator-activated receptor α (*PPARα*), nuclear factor of kappa light polypeptide gene enhancer in B-Cells inhibitor α (*NFKBI* α), and mitogen-activated protein kinase 3 (*MAPK3*) in the skeletal muscle of women with PCOS after a single bout of aerobic exercise [158] was observed. The international evidence-based guidelines for PCOS advise 150–300 min per week of moderate-intensity exercise or 75–150 min per week of vigorous-intensity exercise for the prevention of weight gain and the maintenance of health for women with PCOS [159]. A different study [160] indicated that high-intensity interval training (HIIT) may offer more favourable metabolic benefits, such as improvement in HOMA-IR and lower BMI, compared to lower intensity exercise for women with PCOS, as well as improving mitochondrial functions [36]. Recently, there has been increasing interest in ‘exerkines’, molecules that are released into circulation in response to exercise, transmitting the health impacts of physical activity. In the near future, bioengineered exerkines such as meterorin and irisin will serve as an ideal substitute for individuals incapable of exercise or those who exhibit the limited or non-existent expression of uncoupling protein (UCP1), such as PCOS patients [158].

### 7.2. Diet

Caloric restriction (CR) has proved to be the most efficacious and reproducible dietary intervention to increase healthy lifespan and ageing [161]. It is effective in improving insulin sensitivity [162,163] with over 40% with just 6 months of practice [164] by improving ß-cell function and reducing increased amounts of glucose and HbA1c [9]. It also ameliorates obesity and has also been reported to improve mitochondrial function by activating SIRT1 activator resveratrol that increases mitochondrial content, improving IR [25,165,166]. In recent years, there has been another more sustainable strategy than CR, known as time-restricted eating (TRE), which only limits the eating window without compromising on food consumption. TRE is reported to address mitochondrial dysfunction, IR and hyperandrogenaemia in women with PCOS. It is shown to upregulate genes linked to AMPK signalling, TCA cycle, and ETC [167]. Additionally, one month of maintaining a low-carbohydrate diet also elevated FSH and SHBG levels, thereby reducing IR, when compared to metformin [168]. Furthermore, the “dietary approaches to stop hypertension” (DASH) diet, containing minimal saturated fat, cholesterol, red and processed meats, and refined grains and sweets, but being rich in fruits, vegetables, whole grains, nuts, legumes, and fat-free/low-fat dairy [169], is reported to enhance insulin sensitivity and maintain glycemia, providing both long- and short-term benefits to women with PCOS [9]. In addition, low glycaemic index (GI)-based diets and high-fibre-based diets have also been reported to lower blood glucose and improve insulin sensitivity [170,171]; these results are supported by studies that dietary fibre consumption is conversely linked to fasting insulin, HOMA-IR, and the Matsuda insulin index [172,173]. Unfortunately, dietary changes often prove ineffective in the long term, mirroring the outcomes observed with anti-obesity medications. This may stem from the fact that female participants generally regain weight and struggle to maintain a normal BMI [168]. In recent years, another promising therapeutic target/biomarker for PCOS is advanced glycation end-products (AGEs). According to a one-year-long randomised controlled trial [174], a diet low in AGEs improves IR in obese patients with MetS, suggesting its plausibility in increasing insulin sensitivity and reducing hyperandrogenaemia and inflammation [78].

### 7.3. Therapeutic Agents

For adolescents diagnosed with PCOS, the first line of therapy has conventionally been combined hormonal contraceptives (CHCs), containing oestrogen (ethinyl oestradiol) and progestin. CHCs regulate menstrual cycles, resulting in predictable periods [175]. However, in women with PCOS with cardiovascular and metabolic risk factors, the excess oestrogenic components of CHCs worsen IR, although this effect is compensated by progestin [176]. In addition to CHCs, the growth hormone (GH) is another popular choice which is traditionally administrated to patients with infertility and disordered ovulation [177]. GH treatment lowers the oxidative stress index (OSI), the total oxidant status (TOS), and MMP and results in an over 50% reduction in apoptosis [178] by decreasing the levels of FOXO1, BAX, and caspases 3 and 9. It also diminishes mitochondrial dysfunction by increasing PI3K/AKT and BCL-2 [156].

Metformin, a drug targeting mitochondrial function is used as second-line treatment against PCOS along with ovarian laparoscopic surgery as is lowers the risk of ovarian hyperstimulation syndrome by stimulating exogenous gonadotropin [179,180]. In addition, it was found that metformin inhibits mitochondrial glycerophosphate dehydrogenase, a novel IR therapeutic target, suppressing gluconeogenesis [181]. Moreover, metformin administration in PCOS patients [178] results in decreased leptin levels, a hormone that regulates energy balance, and elevated adiponectin, a hormone that regulates glucose and fatty acid metabolism, providing synergistic activity [182]. This marks metformin as strong candidate for reducing the susceptibility to IR, T2D, and MetS in women with PCOS [107,156]. It has been observed that metformin reduces levels of proinflammatory cytokine IL-6 and CRP [156]. Although it delays the progression of glucose intolerance in women with PCOS, there is no impact on fasting glucose and lipids and other anthropometric parameters [107]. However, moderate weight loss has been observed in PCOS patients on metformin; it has been suggested that metformin be implemented on a long-term basis [183]. In comparison, GLP-1 receptor agonists such as exenatide and liraglutide result in modest weight loss women with PCOS by reducing inflammatory cytokines and improve sex hormone abnormalities, insulin sensitivity, and the menstrual cycle to even greater extent than metformin [184].

In addition to metformin, there are very few insulin-sensitizing drugs that have undergone clinical trials for use in women with PCOS. The ones that have are thiazolidinediones (TZDs), specifically pioglitazone and rosiglitazone, which enhance insulin sensitivity, reduce hyperandrogenaemia, and regulate the menstrual cycle in women with PCOS, acting through the activation of the peroxisome proliferator-activated receptor–gamma (PPARγ), a gene responsible for mitochondrial biogenesis. Furthermore, the administration of pioglitazone in metformin-resistant women with PCOS alleviates metabolic and hormonal abnormalities significantly, suggesting that a combination of drugs is more effective for the management of PCOS in women with more adverse phenotypes [107]. Several studies have also highlighted the role of myo-inositol (MYO) and D-chiro-inositol (DCI) as insulin sensitisers, improving metabolic and oxidative imbalances in women with PCOS [185,186]. Studies suggest that a MYO/DCI ratio of 40:1 is effective in treating women with PCOS, particularly when administered along with metformin or oral contraceptives, resulting in synergistic actions that mitigate severe effects. Moreover, MYO also acts as a secondary messenger in FSH signalling in the ovary, improving ovarian function. However, DCI supplementation could exacerbate insulin-mediated androgen production and fertility in women with PCOS [176,186]. Sodium glucose co-transporter type 2 inhibitor (SGLT2-i) and incretin mimetics are other insulin sensitisers with even stronger impacts on comorbidities such as obesity and CVD. SGLT2-i exhibits cardiovascular and nephron-protective effects by blocking glucose reuptake in the renal proximal tubule, leading to reduced arterial pressure and body weight [187]. Furthermore, SGLT2-i improves BMI and other anthropometric parameters in women with PCOS [188].

Besides insulin sensitisers, letrozole and anti-androgenic drugs have also shown effectiveness in improving IR in women with PCOS. Letrozole, an aromatase inhibitor, is a first-line pharmacological treatment for ovulation induction in infertile anovulatory women with PCOS [159]. A study found that combined letrozole and metformin treatment improved insulin sensitivity by lowering HOMA-IR, insulin levels, and lipid profiles in infertile women with PCOS, while also enhancing ovarian function [189]. Drugs, cyproterone acetate, spironolactone, finasteride, and flutamide are administered to treat hyperandrogenism [176] and IR in women with PCOS and/or hyperandrogenism [190].

### 7.4. Phytochemicals

There are several natural compounds applicable for the treatment of PCOS. For example, berberine, a plant alkaloid, improves insulin action in humans and rodents, by inhibiting the complex I of ETC and subsequent AMPK activation [25]. A decoction made from the roots of *Polygonum multiflorum*, known as Shouwu Jiangqi, has been found to modulate the insulin signalling pathway in rat models of PCOS [191]. Phytosterols in aloe vera can influence the steroidogenic response and induce the expression of oestrogen receptor protein, leading to a reduction in androgen levels and an increase in oestrogen levels, thereby alleviating PCOS symptoms. Moreover, aloe vera also has insulin sensitizing property effecting pancreatic beta cell function. The significant impact of cinnamon extract in improving insulin sensitivity was demonstrated by decreasing fasting blood sugar and IR in women with PCOS [192,193]. Silymarin, a flavonoid extracted from *Silybum marianum* L. *Gaernt*, also has anti-angiogenetic properties, enabling a reduction in follicular cell proliferation, thereby reducing testosterone levels and increasing SHBG protein synthesis and progesterone hormone levels in the corpus luteum. It also affects glucose 6-phosphate and inhibits gluconeogenesis and OS in women with PCOS [15].

### 7.5. Antioxidants

Vitamin D deficiency is associated with IR, infertility, altered SHBG and testosterone levels, and compromised lipid metabolism, factors which are also observed in PCOS; lower vitamin D levels exacerbate PCOS symptoms [10]. In a further study on a DHEA-induced PCOS rat model, vitamin D supplementation reduced obesity, body weight, and uterine and ovarian morphology [194]. Moreover, vitamin D has a positive influence on mitochondria as it upregulates the expression of *TFAM* and the mtDNA copy number, reduces ROS production, and strengthens MMP integrity [195,196]. Meanwhile, other natural antioxidants such as vitamin C and E are incapable of scavenging ROS as they are unable to bind to mitochondria. MitoQ10, a lipophilic cation, can easily pass through the phospholipid bilayer and mitochondrial membrane, then lodges at matrix surface, combating ROS and thus preventing OS and mitochondrial dysfunction. This is supported by a study on rats where PCOS-associated IR was treated with MitoQ10, resulting in an observed decrease in ROS levels, along with increases in MMP and ATP levels [197].

Another notable intervention involves increasing intracellular NAD^+^ levels, as insufficient NAD^+^ levels in the oocytes of women with PCOS may affect follicle/oocyte development. The supplementation of NAD^+^ using nicotinamide riboside (NR) is known to restore NAD^+^ levels and improve IR and mitochondrial functions in the GCs of PCOS women undergoing IVF treatment [198,199].

### 7.6. Mitochondrial Peptides

Mitochondrial peptides such as humanin, a mitochondrial open reading frame of 12S rRNA-c (MOTS-c), and small humanin-like proteins (SHLPs), which maintain mitochondrial function and viability [200], exhibit potential as novel therapeutic agents for PCOS. Humanin, discovered in 2001, is a 24-amino acid cytoprotective peptide [201] that can act as an antioxidant by restoring glutathione levels and enhancing mitobiogenesis [201]. It can also influence insulin secretion, thus enabling glucose uptake [202,203]. In a further study [204], PCOS-induced rats supplemented with the humanin analogue S14G (HNG) showed decreased fasting insulin and blood glucose levels, along with the upregulation of IRS1, AKT, and GLUT4, key proteins in insulin signalling. MOTS-c, a 16 amino acid peptide, increases AMPK and insulin sensitivity, while promoting *NRF2* antioxidant genes. It also regulates mitochondrial insulin levels and homeostasis [205]. Moreover, SHLP-6, -2, and -3 also increase oxygen consumption rate by reducing apoptosis and ROS production [206,207].

### 7.7. Sleep and Mental Health Management

There are reports of association between depression and PCOS-related factors, such as hyperandrogenaemia, IR, and obesity, that warrant the exploration of potential therapies of PCOS-centred depression [208]. A proper sleeping schedule lowers the risk of CVD and other comorbidities linked with PCOS, suggesting is potential in reducing PCOS symptoms [168]. Melatonin, a hormone primarily secreted by the pineal gland and synthesised and metabolised in mitochondria, regulates the sleep cycle by reducing the time required to fall asleep, thereby improving sleep quality. It can also be combined with other therapeutic agents, such as insulin sensitisers and contraceptives, to reduce erratic sleep patterns, stabilise moods, improve insulin sensitivity, and decrease OS, ultimately enhancing the overall quality of life for women with PCOS [209]. Significant associations have been reported between the presence of OSA and fasting glucose or insulin resistance measured by HOMA-IR among women with PCOS, and potential underlying mechanisms include sympathetic overactivity, lipid metabolism alterations, oxidative stress and mitochondrial dysfunction, inflammation, and endothelial dysfunction, leading to diabetes and cardiovascular diseases in these women [210]. The dysregulated transcription of hypoxia after eight weeks of continuous positive airway pressure (CPAP) treatment for OSA in young and morbidly obese PCOS women improved metabolic and cardiovascular outcomes, resulting in modest improvements in insulin sensitivity after controlling for BMI, as well as reductions in the markers of sympathetic and diastolic blood pressure [210,211]. These beneficial effects increase with longer hours of CPAP use and have decreasing effects in women with PCOS with higher degrees of obesity [210]. Dysregulated hypoxia-inducible factor-1 (HIF-1) and HIF-2, members of the HIF family of transcriptional activators increase ROS, decrease antioxidant enzymes and mediate NOX2 gene activation; these are suggested to be molecular mechanisms underlying hypertension, T2D, and cognitive issues stemming from OSA-induced intermittent hypoxia [212].

### 7.8. Mitochondrial Transfer: Future Therapeutic Approach in IVF

Autologous mitochondrial transfer using ovarian stem cells or GCs, as well as the heterologous transfer of isolated mitochondria, represents a promising therapeutic strategy for infertile patients. The autologous approach has the advantage of avoiding heteroplasmy unlike in the heterologous approach. It has been demonstrated that transferring mitochondria isolated from GCs can improve embryo quality and increase pregnancy rate. Similarly, the transfer of mitochondria extracted from oogonial stem cells to mature oocytes has been shown to enhance the rate of high-quality embryos and successful embryo transfers, without altering the maternal mitochondrial genome in cases of recurrent IVF failure [213]. This method could also benefit women with IR who have compromised mitochondria. Additionally, recent research has highlighted the protective effects of mesenchymal stem cell (MSC) transplantation, possibly through the transfer of their own mitochondria, which improves ovarian mitochondrial function, redox status, and IR in a PCOS animal model [214]. These findings suggest that mitochondrial transfer could be a promising therapeutic approach for obese, insulin-resistant women with PCOS undergoing assisted reproductive technology (ART).

## 8. Conclusion and Future Perspectives

While numerous studies associate IR with PCOS and highlight the role of mitochondrial dysfunction in women with PCOS, a missing link connecting these components persists for understanding its role in the development of PCOS. This review aims to bridge the gap by synthesizing current empirical findings. Hyperandrogenism emerges as a crucial factor, driven by elevated LH levels that stimulate androgen secretion from ovarian theca cells. IR and hyperinsulinemia further exacerbate androgen production and impair insulin signalling and GLUT4 translocation, affecting glucose and lipid metabolism. Impaired lipid oxidation hampers mitochondrial function, generating OS and promoting ROS production, which reduces mtDNA copy number, a biomarker of mitochondrial dysfunction in PCOS. Furthermore, IR also predisposes women to comorbidities like T2D and other metabolic complications. The relationship between these factors is not linear but an intricate network forming a vicious cycle. Although more studies are needed in IR and women with PCOS for confirmation, therapeutic interventions targeting multiple key points may more effectively mitigate metabolic complications in PCOS. Exercise, diet, antioxidants and CHCs remain primary defences, but novel interventions such as mitochondrial peptides, AGE-based diets, and antioxidants like MitoQ10 are being explored. Furthermore, clinical trials using mitochondria transfer techniques are required to improve ART outcome in PCOS patients who are obese/insulin resistant. Understanding the interplay between IR, PCOS, and mitochondrial dysfunction can lead to more holistic management strategies for PCOS and related comorbidities.

## Data Availability

Not applicable.

## Abbreviations

The following abbreviations are used in this manuscript:

PCOS	Polycystic Ovary Syndrome
IR	Insulin Resistance
T2D	Type 2 Diabetes
CVD	Cardiovascular Disease
EC	Endometrial Cancer
LH	Luteinizing Hormone
FSH	Follicle-Stimulating Hormone
SHBG	Sex Hormone-Binding Globulin
ROS	Reactive Oxygen Species
OS	Oxidative Stress
mtDNA	Mitochondrial DNA
ETC	Electron Transport Chain
OXPHOS	Oxidative Phosphorylation
PGC-1α	Peroxisome Proliferator-Activated Receptor Gamma Coactivator 1-alpha
NRF-1	Nuclear Respiratory Factor 1
NRF-2	Nuclear Respiratory Factor 2
ERR-α	Oestrogen-Related Receptor Alpha
TFAM	Transcription Factor A, Mitochondrial
Mfn1/2	Mitofusin 1/Mitofusin 2
Opa1	Optic Atrophy 1
DRP1	Dynamin-Related Protein 1
Fis1	Mitochondrial Fission 1 Protein
DAG	Diacylglycerol
CER	Ceramide
PKC	Protein Kinase C
AKT	Protein Kinase B
IRS1	Insulin Receptor Substrate 1
GLUT4	Glucose Transporter Type 4
FFAs	Free Fatty Acids
PA	Palmitate
AIF	Apoptosis-Inducing Factor
NOX4	NADPH Oxidase 4
MCP-1	Monocyte Chemoattractant Protein-1
PTP1B	Protein Tyrosine Phosphatase 1B
JNK	c-Jun N-terminal Kinase
NF-κB	Nuclear Factor Kappa B
MAPK	Mitogen-Activated Protein Kinase
FOXO1	Forkhead Box Protein O1
HMOX1	Heme Oxygenase 1
NOX2	NADPH Oxidase 2
RAC1	Ras-Related C3 Botulinum Toxin Substrate 1
P47PHOX	47 kDa Subunit of NADPH Oxidase
CaMKII	Calcium/Calmodulin-Dependent Protein Kinase II
Myo1c	Myosin-1c
MAMs	Mitochondria-Associated ER Membranes
mTOR	Mammalian Target of Rapamycin
AMPK	AMP-Activated Protein Kinase
SIRT1	Sirtuin 1
DNMT1	DNA Methyltransferase 1
PSEN2	Presenilin 2
COX-1	Cytochrome c Oxidase Subunit 1
MPO	Myeloperoxidase
SDHD	Succinate Dehydrogenase Complex Subunit D
UCRC	Ubiquinol–Cytochrome c Reductase Core Protein
COX7C	Cytochrome c Oxidase Subunit 7C
GLUT1	Glucose Transporter Type 1
GLUT3	Glucose Transporter Type 3
iPSC	Induced Pluripotent Stem Cell
ND1	NADH Dehydrogenase 1
ND2	NADH Dehydrogenase 2
8-OHDG	8-Hydroxy-2′-Deoxyguanosine
ND5	NADH Dehydrogenase 5
LHON	Leber’s Hereditary Optic Neuropathy
TFAM	Transcription Factor A, Mitochondrial
NRF-1	Nuclear Respiratory Factor 1
PPARα	Peroxisome Proliferator-Activated Receptor Alpha
NFKBI α	Nuclear Factor of Kappa Light Polypeptide Gene Enhancer in B-Cells Inhibitor Alpha
MAPK3	Mitogen-Activated Protein Kinase 3
SIRT1	Sirtuin 1
GLUT4	Glucose Transporter Type 4
HNG	Humanin Analogue S14G
TOS	Total Oxidant Status
BCL-2	B-Cell Lymphoma 2
SGLT2-i	Sodium-Glucose Cotransporter 2 Inhibitor
MYO	Myo-Inositol
DCI	D-Chiro-Inositol
MOTS-c	Mitochondrial Open Reading Frame of 12S rRNA-c
SHLP	Small Humanin-Like Protein
ART	Assisted Reproductive Technology
MSC	Mesenchymal Stem Cell
NR	Nicotinamide Riboside
SHBG	Sex Hormone-Binding Globulin
OSA	Obstructive Sleep Apnoea
